# *Helicobacter pylori* Antibody Reactivities and Colorectal Cancer Risk in a Case-control Study in Spain

**DOI:** 10.3389/fmicb.2017.00888

**Published:** 2017-05-29

**Authors:** Nerea Fernández de Larrea-Baz, Angelika Michel, Beatriz Romero, Beatriz Pérez-Gómez, Victor Moreno, Vicente Martín, Trinidad Dierssen-Sotos, José J. Jiménez-Moleón, Jesús Castilla, Adonina Tardón, Irune Ruiz, Rosana Peiró, Antonio Tejada, María D. Chirlaque, Julia A. Butt, Rocío Olmedo-Requena, Inés Gómez-Acebo, Pedro Linares, Elena Boldo, Antoni Castells, Michael Pawlita, Gemma Castaño-Vinyals, Manolis Kogevinas, Silvia de Sanjosé, Marina Pollán, Rosa del Campo, Tim Waterboer, Nuria Aragonés

**Affiliations:** ^1^Environmental and Cancer Epidemiology Area, National Center of Epidemiology, Instituto de Salud Carlos IIIMadrid, Spain; ^2^Consortium for Biomedical Research in Epidemiology and Public Health (CIBER of Epidemiology and Public Health) – Centro de Investigación Biomédica en Red de Epidemiología y Salud Pública (CIBERESP)Madrid, Spain; ^3^Division of Molecular Diagnostics of Oncogenic Infections, Infection, Inflammation and Cancer Program, German Cancer Research Center (DKFZ)Heidelberg, Germany; ^4^Department of Microbiology, Ramón y Cajal University Hospital (IRYCIS)Madrid, Spain; ^5^Cancer Epidemiology Research Group, Oncology and Hematology Area, IIS Puerta de Hierro (Puerta de Hierro Health Research Institute)Madrid, Spain; ^6^Cancer Prevention and Control Program, Catalan Institute of OncologyHospitalet de Llobregat, Spain; ^7^Department of Clinical Sciences, Faculty of Medicine, University of BarcelonaBarcelona, Spain; ^8^Colorectal Cancer Group, Bellvitge Biomedical Research Institute (IDIBELL)Hospitalet de Llobregat, Spain; ^9^The Research Group in Gene – Environment and Health Interactions, University of LeónLeón, Spain; ^10^Area of Preventive Medicine and Public Health, Faculty of Health Sciences, Department of Biomedical Sciences, University of LeónLeón, Spain; ^11^Division of Epidemiology and Computational Biology, School of Medicine, University of Cantabria-IDIVALSantander, Spain; ^12^Granada Health Research Institute (ibs.GRANADA) – Instituto de Investigación Biosanitaria de GranadaGranada, Spain; ^13^Department of Preventive Medicine and Public Health, University of GranadaGranada, Spain; ^14^Instituto de Salud Pública de Navarra, IdiSNA-Navarra Institute for Health ResearchPamplona, Spain; ^15^Molecular Epidemiology of Cancer Unit, Oncology Institute, Department of Medicine, University of OviedoOviedo, Spain; ^16^Department of Pathology, Donostia University HospitalDonostia, Spain; ^17^Foundation for the Promotion of Health and Biomedical Research of Valencia Region (FISABIO) – Fundación para el Fomento de la Investigación Sanitaria y Biomédica de la Comunitat Valenciana FISABIO–Salud PúblicaValencia, Spain; ^18^Coloproctology Unit, Department of General Surgery, Huelva University Hospital ComplexHuelva, Spain; ^19^Department of Epidemiology, Regional Health Council, IMIB-ArrixacaMurcia, Spain; ^20^Department of Health and Social Sciences, University of MurciaMurcia, Spain; ^21^Department of Gastroenterology and Hepatology, Complejo Asistencial Universitario de LeónLeón, Spain; ^22^Gastroenterology Department, Hospital ClínicBarcelona, Spain; ^23^Institut d'Investigacions Biomèdiques August Pi i Sunyer (IDIBAPS)Barcelona, Spain; ^24^CIBER Liver and Digestive Diseases – CIBER Enfermedades Hepáticas y Digestivas (CIBEREHD)Madrid, Spain; ^25^Department of Gastroenterology, University of BarcelonaBarcelona, Spain; ^26^ISGlobal, Centre for Research in Environmental Epidemiology (CREAL)Barcelona, Spain; ^27^Hospital del Mar Medical Research Institute (IMIM)Barcelona, Spain; ^28^Department of Experimental and Health Sciences, Universitat Pompeu FabraBarcelona, Spain; ^29^Cancer Epidemiology and Research Program, Catalan Institute of Oncology-IDIBELLHospitalet de Llobregat, Spain; ^30^Spanish Network for Research in Infectious Diseases – Red Española de Investigación en Patología InfecciosaSevilla, Spain

**Keywords:** *Helicobacter pylori*, multiplex serology, colorectal neoplasm, chronic infection, bacterial infections, non-infectious diseases

## Abstract

**Background:** Several studies have suggested that *Helicobacter pylori* (*H. pylori*) infection is a risk factor for colorectal cancer (CRC), while others have not confirmed this hypothesis. This work aimed to assess the relation of CRC with *H. pylori* seropositivity and with seropositivity to 16 *H. pylori* proteins, in the MultiCase-Control study, MCC-Spain.

**Methods:** MCC-Spain is a multicase-control study carried out in Spain from 2008 to 2013. In total, 2,140 histologically-confirmed incident CRC cases and 4,098 population-based controls were recruited. Controls were frequency-matched by sex, age, and province. Epidemiological data were collected through a questionnaire fulfilled by face-to-face interviews and a self-administered food-frequency questionnaire. Seroreactivities against 16 *H. pylori* proteins were determined in 1,488 cases and 2,495 controls using *H. pylori* multiplex serology. *H. pylori* seropositivity was defined as positivity to ≥4 proteins. Multivariable logistic regression mixed models were used to estimate odds ratios (OR) and 95% confidence intervals (CI).

**Results:** *H. pylori* seropositivity was not associated with increased CRC risk (OR = 0.91; 95% CI: 0.71–1.16). Among *H. pylori* seropositive subjects, seropositivity to Cagδ showed a lower CRC risk, and risk decreased with increasing number of proteins seropositive. Seropositivity to the most recognized virulence factors, CagA and VacA, was not associated with a higher CRC risk. No statistically significant heterogeneity was identified among tumor sites, although inverse relations were stronger for left colon cancer. An interaction with age and sex was found: *H. pylori* seropositivity was associated with a lower CRC risk in men younger than 65 and with a higher risk in older women.

**Conclusions:** Our results suggest that neither *H. pylori* seropositivity, nor seropositivity to the virulence factor CagA are associated with a higher CRC risk. A possible effect modification by age and sex was identified.

## Introduction

*Helicobacter pylori* (*H. pylori*) is the *Helicobacter* species that predominantly infects humans. According to the usual site of colonization, *Helicobacter* species can be divided into gastric and enteric or enterohepatic *Helicobacter* types (International Agency for Research on Cancer, 2012). Though most literature on the implication of *H. pylori* in the etiopathogenesis of cancer refers to gastric cancer [adenocarcinoma and low-grade B-cell mucosa-associated lymphoid tissue (MALT) gastric lymphoma], there are also studies investigating its role in cancer of other organs of the digestive system, including esophagus, colon and rectum, pancreas, and biliary tract (Siddheshwar et al., 2001; Trikudanathan et al., 2011; Sonnenberg and Genta, 2013; Xiao et al., 2013; Murphy et al., 2014; Wang et al., 2014; Chen et al., 2015), and even of extra-digestive organs, such as lung or larynx (Rezaii et al., 2008; Mounika, 2013).

Regarding a possible association between *H. pylori* infection and colorectal cancer (CRC) risk, there are no consistent results in the scientific literature. Several meta-analyses (Zumkeller et al., 2006; Zhao et al., 2008; Hong et al., 2012; Chen et al., 2013; Rokkas et al., 2013; Wu et al., 2013; Guo and Li, 2014; Liu and Zheng, 2016) have obtained combined odds ratios (OR) over the unity (range from 1.08 to 1.63), suggesting an increased CRC risk associated with *H. pylori* infection. However, heterogeneity among studies and insufficient control for confounding factors in most of them entail a high degree of uncertainty, which precludes from deriving solid conclusions. Biological plausibility has been investigated and several mechanisms have been proposed to explain an increased risk of CRC due to *H. pylori* infection. The most established involve the increase of gastrin secretion, the modification of gut microbiota and the chronic inflammation status (Chang and Parsonnet, 2010; Tatishchev et al., 2012; Papastergiou, 2016). However, two recent publications, one reporting results from a nested case-control study including a Caucasian population from the US (Blase et al., 2016) and another from a cohort study in Germany (Chen et al., 2016), not included in the above mentioned meta-analyses, did not find a statistically significant association between *H. pylori* infection and CRC.

The pathogenicity of different *H. pylori* strains colonizing the gastric mucosa has been involved in modulating the risk of gastric adenocarcinoma. Whether such an effect also exists for CRC has been studied to a lesser extent, but could be one of the factors contributing to heterogeneity among the studies' results. *H. pylori* multiplex serology is a recently developed technique able to quantify seroreactivity against several *H. pylori* proteins in a wide set of serum samples in a single assay. It therefore allows obtaining a detailed characterization of the serological response against *H. pylori*, as a surrogate marker of differences in bacterial protein expression patterns, in large population samples.

The aims of this study are to evaluate the association between *H. pylori* seropositivity as well as seropositivity against 16 individual *H. pylori* proteins and CRC risk. CRC cases and controls of the MCC-Spain study were examined, controlling for the main potential confounding factors and exploring differences among cancer sites, age groups, and sex.

## Materials and methods

### Study population

We used data from the participants in the MCC-Spain multicase-control project, a large multicenter study with population-based controls. This study aimed to investigate environmental and genetic factors involved in the etiology of various forms of cancer. Following a standardized protocol, patients with a new diagnosis of gastric, colorectal, breast, or prostate cancer, and chronic lymphocytic leukemia cases, aged 20–85 years, were invited to participate in 23 hospitals from 12 Spanish geographical regions (provinces). All cases had lived in the catchment area of each hospital for at least 6 months prior to diagnosis. Each province recruited at least two different cancer types. In parallel, a single group of population-based controls was randomly selected from the general population living in the catchment areas of the collaborating hospitals, frequency-matched for age and sex to the whole set of cases included in each province. Recruitment started in September 2008 and lasted until December 2013, though the period of recruitment differed by hospital. Of the 23 hospitals, 18 from 11 Spanish provinces recruited CRC cases: Asturias, Barcelona, Cantabria, Gipuzkoa, Granada, Huelva, León, Madrid, Murcia, Navarra, and Valencia. The Ethical Review Board of each participating center approved the study protocol. All participants provided written informed consent for their enrolment in the study. More details regarding the design of the study are provided elsewhere (Castaño-Vinyals et al., 2015).

### Data collection

Experienced interviewers conducted structured interviews to cases and controls, to collect information on socio-demographic factors, life-styles, weight and height at various periods of life, occupational history, medical history, and family history of cancer. Dietary habits were obtained through a food-frequency questionnaire provided to each participant at the interview for self-fulfillment and returned by mail. Following the study protocol, all cases and controls were asked to donate a blood sample. Specimens were refrigerated locally until being processed and aliquoted (in <48 h). Then, they were stored at –80°C until they were sent to the laboratory.

**Figure 1:**
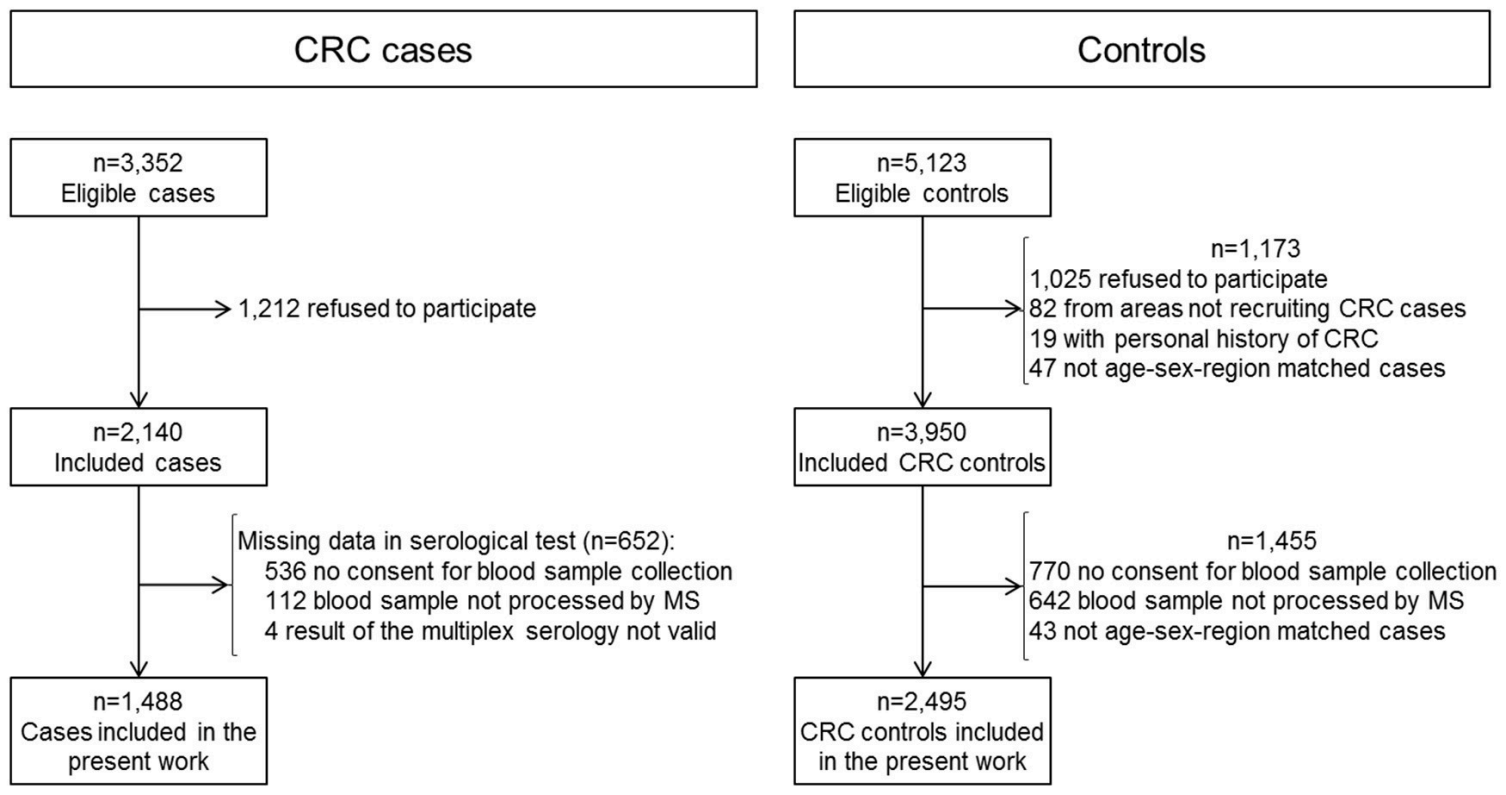
**Flow of colorectal cancer cases and controls through the MCC-Spain study stages**. CRC, Colorectal cancer; MS, Multiplex serology.

In total, 2,140 histopathologically confirmed CRC cases and 3,950 controls were included. The analysis presented here is based on 1,488 (70%) CRC cases and 2,495 (63%) controls. Main reasons for not being included in the analysis were not having donated a blood sample and sample not having been processed by multiplex serology (Figure 1).

### Laboratory assays

Seroreactivities against 16 *H. pylori* proteins were determined using *H. pylori* multiplex serology (Supplementary Table 1). Multiplex serology is a glutathione S-transferase (GST) capture immunosorbent assay combined with fluorescent-bead technology, as described elsewhere (Waterboer et al., 2005). This technique simultaneously quantifies antibodies directed against an array of antigens. In brief, bacterially expressed recombinant GST-*H. pylori* fusion proteins were used as antigens. The fusion proteins were loaded and affinity-purified directly on individual sets of spectrally distinct glutathione-casein-coupled fluorescence-labeled polystyrene beads (SeroMap, Luminex, Austin, TX). Bead sorts, each carrying a different antigen, were mixed and incubated with human sera at 1:100 dilutions. Antibodies bound to the beads via the bacterial antigens were stained by biotinylated anti-human-IgA, IgM, IgG (Dianova, Hamburg, Germany), and streptavidin-R-phycoerythrin. Beads were examined in a Luminex 200 analyzer that quantifies the antibody bound to bacterial antigen via the median R-phycoerythrin fluorescence intensity of at least 100 beads of the same internal color. Net (bead and GST background subtracted) median reporter fluorescence intensity (MFI)-values were calculated and negative values were set to +1.

For *H. pylori* proteins, serostatus cut-offs were calculated (mean MFI + 3 SD, excluding positive outliers) in 17 *H. pylori* negative sera previously classified for *H. pylori* status run within the same experiment. According to these cut-offs (Supplementary Table 1), each participant was classified as seropositive or seronegative to each protein. Following previously published criteria, *H. pylori* seropositivity was defined as positivity for at least four of the 15 *H. pylori* proteins (excluding HomB, a protein recently added to *H. pylori* multiplex serology). Subjects fulfilling this criterion were considered infected (Michel et al., 2009).

### Statistical analysis

Cases and controls characteristics were summarized by frequencies and percentages for categorical variables, and by means and standard deviations for continuous variables. To test for possible differences among cases and controls, and among infected and non-infected controls in the distribution of potential confounding factors, univariate (chi-squared or Mann-Whitney U-test for categorical and continuous variables, respectively) and multivariable (logistic regression) analyses were done.

Multivariable logistic regression mixed models were used to quantify the association between *H. pylori* seropositivity and CRC risk, estimating ORs and their 95% confidence intervals (CI). First a basic model (model 1) was built, that was adjusted for gender, age (as a continuous variable), and education. Secondly, a model including potential confounders such as smoking status, body mass index (BMI), and family history of CRC was developed (model 2). Lastly a fully adjusted model was done (model 3), that was additionally adjusted by ethanol intake at age 30–40 (g/day) and dietary factors: total energy intake (calories/day), vegetables intake (g/day), and red and processed meat intake (g/day). In all models province was included as a random-effect term.

Among *H. pylori* positive cases and controls, the association between serostatus against each of the 16 proteins and CRC risk was then investigated. Multivariable logistic regression mixed models were used, adjusting by gender, age, education level, smoking status, and CRC family history. As a sensitivity analysis, models additionally adjusted by BMI, ethanol intake at age 30–40 and dietary factors were constructed. Province was included as a random-effect term.

For those proteins whose serostatus resulted independently associated with CRC risk, dose-response relation was assessed by analyzing the association between seroreactivity and CRC risk. Seroreactivity was categorized in tertiles, based on the distribution of the MFI for each protein in infected controls seropositive against that protein.

A possible differential effect by tumor site was analyzed. For this purpose, multinomial logistic regression mixed models were fitted. Heterogeneity of the effects was assessed using a Wald-test comparing the coefficients obtained for the different CRC sites. Tumors located in cecum, ascending colon, hepatic flexure or transverse colon were grouped as right colon cancer, those located in the splenic flexure, descending colon or sigmoid, as left colon and tumors classified as rectosigmoideal were grouped with rectum cancer cases.

To assess the association of seropositivity against each protein independent of serostatus of other proteins, a multivariable logistic regression mixed analysis was carried out simultaneously including all the proteins associated with colorectal, colon, or rectum cancer with a *p* < 0.10 in the individual analyses also adjusting by gender, age, education, smoking status, and CRC family history.

Effect modification by age, sex, and education was explored by comparing models with and without the interaction term and assessed through the likelihood-ratio test. Age was dichotomized using as cut-off value the median of the age in controls. Stratified analyses were performed where interaction was present.

The raw data analyzed for the current study are not publicly available due to confidentiality related restrictions, but they would be available from the last author or from one of the co-principal investigators on reasonable request [Dr. N. Aragonés (naragones@isciii.es)/Dr. M. Pollán (mpollan@isciii.es)].

## Results

Cases of CRC were predominantly men (64%), had a mean age of 67 years (SD: 11) and a low education (72% primary school or lower). Controls were, on average, 3 years younger, with lower history of CRC among their first-degree relatives and had a higher education level (Table 1). With respect to differences in dietary habits, controls had higher intake of vegetables, nuts and smoked foods, lower intake of red and processed meat, and lower overall energy intake. Clinico-pathological characteristics of cases are summarized in Supplementary Table 2. Tumor was located in the colon in 910 cases [402 (27%) right colon, 507 (34%) left colon, and 1 not specified], and in the rectum in 556 cases (37%). For 22 cases (1.5%) colon or rectum location could not be classified. Most tumors were adenocarcinomas (97%).

**Table 1:** **Characteristics of colorectal cancer cases and controls**.

**Variable**	**Controls (*N* = 2,495)**	**Cases^a^**					
		**All (*N* = 1,488)**		**Colon (*N* = 910)**		**Rectum (*N* = 556)**	
	***N* (%)**	***N* (%)**	***p*-values**	***N* (%)**	***p*-values**	***N* (%)**	***p*-values**
Sex			<0.001		<0.001		<0.001
Male	1,275 (51)	945 (64)		552 (61)		376 (68)	
Female	1,220 (49)	543 (36)		358 (39)		180 (32)	
Age (years)^b^	63.6 [11.7]	66.7 [10.6]	<0.001	67.3 [10.6]	<0.001	65.8 [10.6]	<0.001
Race			0.521		0.510		0.192
White/Caucasian	2,451 (98)	1,465 (98)		891 (98)		552 (99)	
Other	42 (2)	23 (2)		19 (2)		4 (1)	
Missing	2 (0)	0 (0)		0 (0)		0 (0)	
Education			<0.001		<0.001		<0.001
No/incomplete primary school	482 (19)	471 (32)		291 (32)		176 (32)	
Primary school	891 (36)	589 (40)		356 (39)		224 (40)	
Secondary school	685 (27)	284 (19)		169 (19)		109 (20)	
University degree	437 (18)	144 (10)		94 (10)		47 (8)	
Smoking status			0.013		0.039		0.023
Never smoker	1,099 (44)	612 (41)		398 (44)		208 (37)	
Former smoker	854 (34)	577 (39)		345 (38)		223 (40)	
Current smoker	533 (21)	289 (19)		161 (18)		122 (22)	
Missing	9 (0)	10 (1)		6 (1)		3 (1)	
Past ethanol intake^c^			<0.001		0.003		0.029
No drinker	611 (24)	377 (25)		235 (26)		136 (24)	
Light	455 (18)	205 (14)		127 (14)		77 (14)	
Upper recommended limit	505 (20)	280 (19)		162 (18)		113 (20)	
Abundant	440 (18)	285 (19)		166 (18)		114 (21)	
Heavy/Very heavy	205 (8)	166 (11)		101 (11)		62 (11)	
Missing	279 (11)	175 (12)		119 (13)		54 (10)	
BMI (kg/m^2^)			<0.001		<0.001		0.008
<25	788 (32)	331 (22)		183 (20)		142 (26)	
25–29.9	930 (37)	540 (36)		324 (36)		211 (38)	
≥30	437 (18)	304 (20)		195 (21)		103 (19)	
Missing	340 (14)	313 (21)		208 (23)		100 (18)	
CRC family history			<0.001		<0.001		<0.001
No CRC family history	2,212 (89)	1,162 (78)		719 (79)		425 (76)	
Only 2nd degree relatives	65 (3)	58 (4)		37 (4)		20 (4)	
≥1 first degree relatives	207 (8)	255 (17)		145 (16)		107 (19)	
Missing	11 (0)	13 (1)		9 (1)		4 (1)	
	**Mean [*****SD*****]**	**Mean [*****SD*****]**	***p*****-values**	**Mean [*****SD*****]**	***p-*****values**	**Mean [*****SD*****]**	***p*****-values**
Diet related variables							
Total energy (cal/d)	1,901.3 [557.4]	2,019.3 [631.3]	<0.001	1,989.1 [611.1]	<0.001	2,061.0 [657.6]	<0.001
Fruits (g/d)	357.0 [217.2]	349.8 [199.0]	0.548	360.0 [200.5]	0.42	337.3 [197.2]	0.071
Vegetables (g/d)	193.8 [122.9]	175.9 [106.3]	<0.001	181.5 [107.8]	0.009	169.1 [104.1]	<0.001
Red/processed meat (g/d)	61.9 [38.1]	74.9 [47.8]	<0.001	72.3 [45.8]	<0.001	78.6 [50.2]	<0.001
Smoked cold meat/fish (g/d)	3.3 [8.4]	2.9 [8.3]	<0.001	2.7 [7.8]	<0.001	3.1 [8.9]	0.003
Nuts (g/d)	7.9 [13.3]	7.1 [12.2]	0.008	7.1 [12.8]	0.003	7.0 [11.4]	0.357
Dairy (g/d)	364.9 [186.1]	366.6 [190.4]	0.995	376.1 [189.9]	0.234	352.8 [191.6]	0.147
Fiber (g/d)	22.9 [9.1]	22.5 [8.2]	0.301	22.5 [8.1]	0.417	22.5 [8.4]	0.606
Calcium (mg/d)	924.8 [306.2]	934.1 [326.6]	0.651	942.4 [326.5]	0.301	924.0 [329.1]	0.702
D vitamin (mcg/d)	2.7 [1.5]	2.8 [1.6]	0.951	2.7 [1.6]	0.717	2.8 [1.7]	0.716
METS 2–12 years before diagnosis	154.8 [263.1]	141.8 [260.6]	<0.001	129.2 [237.3]	<0.001	163.3 [295.9]	0.035

*BMI, Body mass index; CRC, Colorectal cancer; METS, Metabolic Equivalent Units*.

a *For 22 cases site information was not available*.

b *Values represent mean and standard deviations*.

c *Alcohol intake categories (based on g/d of ethanol intake): Light: ≤ 6; Upper recommended limit: women >6 to ≤ 12 and men >6 to ≤ 24; Abundant: women: >12 to ≤ 24 and men >24 to ≤ 60; Heavy/Very heavy: women >24 and men >60. For continuous variables, analysis was done over participants with complete information: 2,216 controls and 1,313 cases for diet variables; 2,494 controls and all cases for physical activity (METS). In the multivariable logistic regression analysis including variables with a p < 0.10 in any of the comparisons in the table, age, education, family history of colorectal cancer, total energy intake, and intakes of vegetables, nuts, red and processed meat, and smoked food were associated with CRC at a p < 0.05*.

Overall, *H. pylori* seropositivity was 88% (95% CI: 86–89) in controls and 90% (95% CI: 88–91) in CRC cases, with no differences by tumor anatomic site. Distribution of potential confounding factors by *H. pylori* serostatus in controls is presented in Supplementary Table 3. Among factors associated with the case/control status in the univariable analysis in our sample, sex, age, education, BMI, alcohol consumption, and intakes of vegetables and nuts appeared associated also with the exposure to *H. pylori* infection in controls. However, after mutually adjusting by factors with a *p* < 0.10 in the univariable analysis, only sex and intake of vegetables were associated with *H. pylori* serostatus using a *p*-value limit of 0.05 (data not shown).

In Table 2 seroprevalence for each *H. pylori* protein among infected controls and cases can be seen. The highest prevalences were detected for GroEL, NapA, HP231, and Omp, and the lowest for HpaA, HomB, and Cad. Infected to non-infected seropositivity ratio were highest for HcpC (20 in controls and 32 in cases) and lowest for HomB (two both, in controls and cases).

**Table 2:** **Seroprevalence for *H. pylori* proteins among infected^a^ controls and colorectal cancer cases**.

	**Controls**	**Cases^b^**		
	**(*N* = 2,186)**	**All (*N* = 1,335)**	**Colon (*N* = 814)**	**Rectum (*N* = 500)**
	***N* (%)**	***N* (%)**	***N* (%)**	***N* (%)**
GroEL+	1,962 (90)	1,234 (92)	757 (93)	459 (92)
NapA+	1,806 (83)	1,138 (85)	692 (85)	429 (86)
HP231+	1,758 (80)	1,055 (79)	637 (78)	401 (80)
Omp+	1,613 (74)	990 (74)	586 (72)	387 (77)
HyuA+	1,440 (66)	906 (68)	553 (68)	338 (68)
VacA+	1,346 (62)	790 (59)	471 (58)	307 (61)
Catalase+	1,308 (60)	760 (57)	464 (57)	287 (57)
UreA+	1,198 (55)	710 (53)	433 (53)	267 (53)
HP305+	1,169 (53)	658 (49)	391 (48)	256 (51)
CagA+	1,168 (53)	743 (56)	465 (57)	269 (54)
CagM+	1,100 (50)	677 (51)	412 (51)	257 (51)
Cagδ+	1,065 (49)	590 (44)	339 (42)	242 (48)
HcpC+	989 (45)	563 (42)	334 (41)	221 (44)
HpaA+	804 (37)	435 (33)	270 (33)	163 (33)
HomB+	779 (36)	456 (34)	279 (34)	173 (35)
Cad+	711 (33)	410 (31)	251 (31)	154 (31)
**Number of proteins**+				
4–7	716 (33)	457 (34)	280 (34)	171 (34)
7–12	1,043 (48)	665 (50)	412 (51)	241 (48)
≥12	427 (20)	213 (16)	122 (15)	88 (18)
Number of proteins+^c^	8.9 [2.8]	8.7 [2.6]	8.7 [2.6]	8.9 [2.6]

a *Positive for 4 or more H. pylori proteins*.

b *For 22 cases site information was not available*.

c *Mean [SD] of the number of proteins against which antibody reactivities were above the corresponding cut-off value*.

As shown in Table 3, in the multivariable analysis *H. pylori* seropositivity was not statistically significantly associated with a higher CRC risk, neither in the basal model, nor in models adjusted by different sets of covariates. An inverse relation between the number of proteins with seroreactivity above the corresponding cut-off level and CRC risk emerged when adjusting by BMI and/or diet and alcohol consumption variables. CRC risk was 5% lower for each additional seropositive protein. By tumor site, *H. pylori* infection tended to be associated with a lower risk of left colon cancer, although result of the statistical test for heterogeneity was not significant.

**Table 3:** **Association between colorectal cancer and *H. pylori* infection**.

		**Colorectal^a^**			**Right colon^b^**			**Left colon^b^**		**Rectum^b^**			
	**OR**	**95% CI**	***p*-values**	**OR**	**95% CI**	***p*-values**	**OR**	**95% CI**	***p*-values**	**OR**	**95% CI**	***p*-values**	***p*-het**
**MODEL 1**													
*H. pylori*+	1.03	0.83–1.29	0.775	1.33	0.90–1.96	0.150	0.84	0.62–1.13	0.244	1.05	0.77–1.44	0.761	0.118
**Number of proteins+**													
<4	1.00			1.00			1.00			1.00			
4–7	1.08	0.85–1.38	0.522	1.35	0.89–2.04	0.164	0.89	0.64–1.24	0.492	1.12	0.79–1.57	0.533	0.231
7–12	1.09	0.86–1.37	0.483	1.45	0.97–2.18	0.069	0.88	0.64–1.22	0.453	1.06	0.76–1.48	0.715	0.114
≥12	0.81	0.62–1.07	0.135	0.99	0.62–1.59	0.981	0.62	0.42–0.92	0.016	0.90	0.61–1.32	0.592	0.178
Trend	0.94	0.87–1.02	0.120	0.99	0.88–1.13	0.914	**0.88**	**0.79–0.98**	**0.026**	0.95	0.86–1.06	0.404	0.264
**MODEL 2**													
*H. pylori*+	0.91	0.71–1.16	0.434	1.14	0.74–1.73	0.556	0.72	0.52–1.01	0.059	0.95	0.68–1.34	0.784	0.168
**Number of proteins+**													
<4	1.00			1.00			1.00			1.00			
4–7	1.00	0.77–1.31	0.972	1.20	0.76–1.90	0.430	0.78	0.54–1.13	0.191	1.10	0.76–1.60	0.615	0.198
7–12	0.96	0.74–1.24	0.739	1.25	0.81–1.95	0.313	0.80	0.56–1.14	0.217	0.93	0.65–1.34	0.709	0.230
≥12	0.63	0.46–0.85	0.003	0.73	0.43–1.25	0.254	0.45	0.29–0.71	0.001	0.76	0.50–1.16	0.200	0.143
Trend	**0.87**	**0.80–0.95**	**0.002**	0.92	0.80–1.06	0.265	**0.82**	**0.72–0.93**	**0.002**	0.89	0.79–1.00	0.058	0.385
**MODEL 3**													
*H. pylori*+	0.87	0.67–1.12	0.276	1.04	0.67–1.62	0.856	**0.67**	**0.47–0.96**	**0.031**	0.95	0.66–1.36	0.768	0.177
**Number of proteins+**													
<4	1.00			1.00			1.00			1.00			
4–7	1.01	0.76–1.34	0.972	1.16	0.72–1.89	0.540	0.77	0.52–1.15	0.204	1.14	0.76–1.69	0.526	0.218
7–12	0.90	0.68–1.18	0.435	1.14	0.71–1.81	0.591	0.73	0.50–1.07	0.104	0.91	0.62–1.34	0.629	0.271
≥12	0.57	0.41–0.80	0.001	0.63	0.36–1.11	0.110	0.39	0.24–0.64	< 0.001	0.73	0.47–1.15	0.176	0.104
Trend	**0.84**	**0.76–0.92**	<**0.001**	0.88	0.75–1.02	0.085	**0.78**	**0.68–0.89**	<**0.001**	**0.87**	**0.76–0.99**	**0.033**	0.324

a *From multilevel logistic regression mixed model*.

b *From multinomial logistic regression mixed model*.

*Province was included as a random-effect term in all models. Model 1: Adjusted by age, sex and education. Model 2: Additionally adjusted by smoking status, body mass index and family history of colorectal cancer. Analysis based on 2,138 controls, 1,160 colorectal, 299 right colon, 394 left colon, and 450 rectal cancer cases with complete information for all the covariates. Model 3: Additionally adjusted by total energy intake, past ethanol intake, vegetables intake and red and processed meat intake. Analysis based on 1,914 controls, 1,031 colorectal, 265 right colon, 342 left colon and 409 rectal cancer cases with complete information for all the covariates. Trend corresponds to the OR for each increasing category of number of proteins seropositive. P-het, p-values for the heterogeneity among tumor sites. Associations with a p < 0.05 are highlighted in bold*.

**Figure 2:**
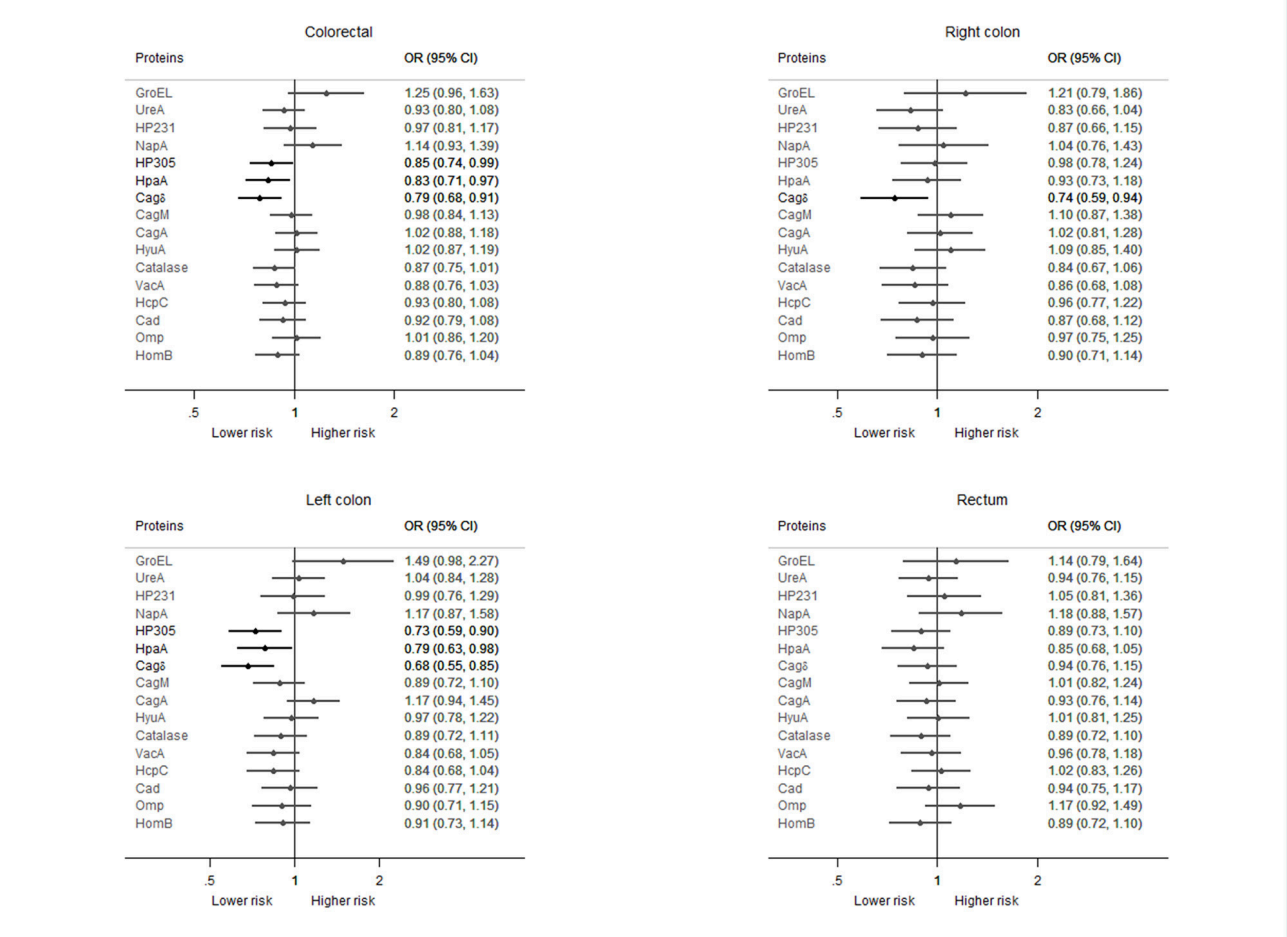
**Association of the seropositivity against each of the studied *H. pylori* proteins with the risk of colorectal cancer among *H. pylori* seropositive participants (positive for four or more *H. pylori* proteins), overall and by tumor site**. ORs from multinomial logistic regression mixed model adjusted by age, sex, education, family history of colorectal cancer, and smoking status; province included as a random-effect term. Statistically significant associations are highlighted in black. Analyses based on 2,476 controls, 1,467 colorectal, 395 right colon, 500 left colon, and 550 rectal cancer cases with complete information for all the covariates.

Among *H. pylori* infected subjects, only seropositivity against GroEL and NapA showed a positive association with CRC risk, although CIs were wide (Figure 2). On the contrary, seropositivity to HP305, HpaA, and Cagδ was related to a 15–21% lower risk of CRC compared to seronegativity against each of these proteins. An inverse relation was observed between the number of seropositivities and CRC risk, showing a 3% lower risk for each seropositive protein. No remarkable differences were apparent in the direction or the magnitude of these associations depending on tumor site. Only the estimated effect of Cagδ showed heterogeneity among sites according to the statistical test. Seropositivity for this protein was associated with a 26 and 32% lower risk of right and left colon cancer, respectively, while only a non-statistically significant 6% lower risk was observed for rectum cancer. Additionally, for HP305 and HpaA the magnitude of the association was stronger (lower ORs) and statistically significant for left colon cancer, although the direction of the estimated ORs were the same (under the unity) for all sites and statistical test for heterogeneity did not reach the significance level (Figure 2). Additional adjustment of the models by BMI, ethanol consumption and diet factors resulted in a stronger inverse association for some of the antigens' serostatus (Supplementary Figure 1).

Adjusting simultaneously by serostatus against all those proteins individually associated with risk (GroEL, HP305, HpaA, Cagδ, and Catalase), only Cagδ remained inversely associated at a statistically significant level with CRC (OR = 0.81; 95% CI: 0.70–0.94). By tumor site, Cagδ seropositivity remained inversely associated with right and left colon cancers (OR = 0.76; 95% CI: 0.60–0.96 and OR = 0.71; 95% CI: 0.57–0.88, respectively) and HP305 only with left colon cancer (OR = 0.75; 95% CI: 0.60–0.94). Seropositivity to GroEL appeared related to a higher CRC (OR = 1.33; 95% CI: 1.01–1.74) and left colon cancer risk (OR = 1.62; 95% CI: 1.06–2.48). For rectal cancer, ORs showed the same directions than for the other sites, but with wider CIs, and none of them was statistically significant. Heterogeneity among tumor sites was nearly statistically significant only for the effect of Cagδ (*p* = 0.055). A dose-response pattern was observed for these associations. Higher seroreactivity against GroEL was associated with increasing CRC risk (8% higher for each tertile with respect to seronegativity; *p* = 0.049), and for Cagδ and HP305 higher seroreactivities were associated with decreasing CRC risk: 11% (*p* < 0.001) and 8% (*p* = 0.011) lower for each tertile with respect to seronegativity, respectively. Results were similar for the three tumor sites analyzed, with no statistically significant heterogeneity.

**Figure 3:**
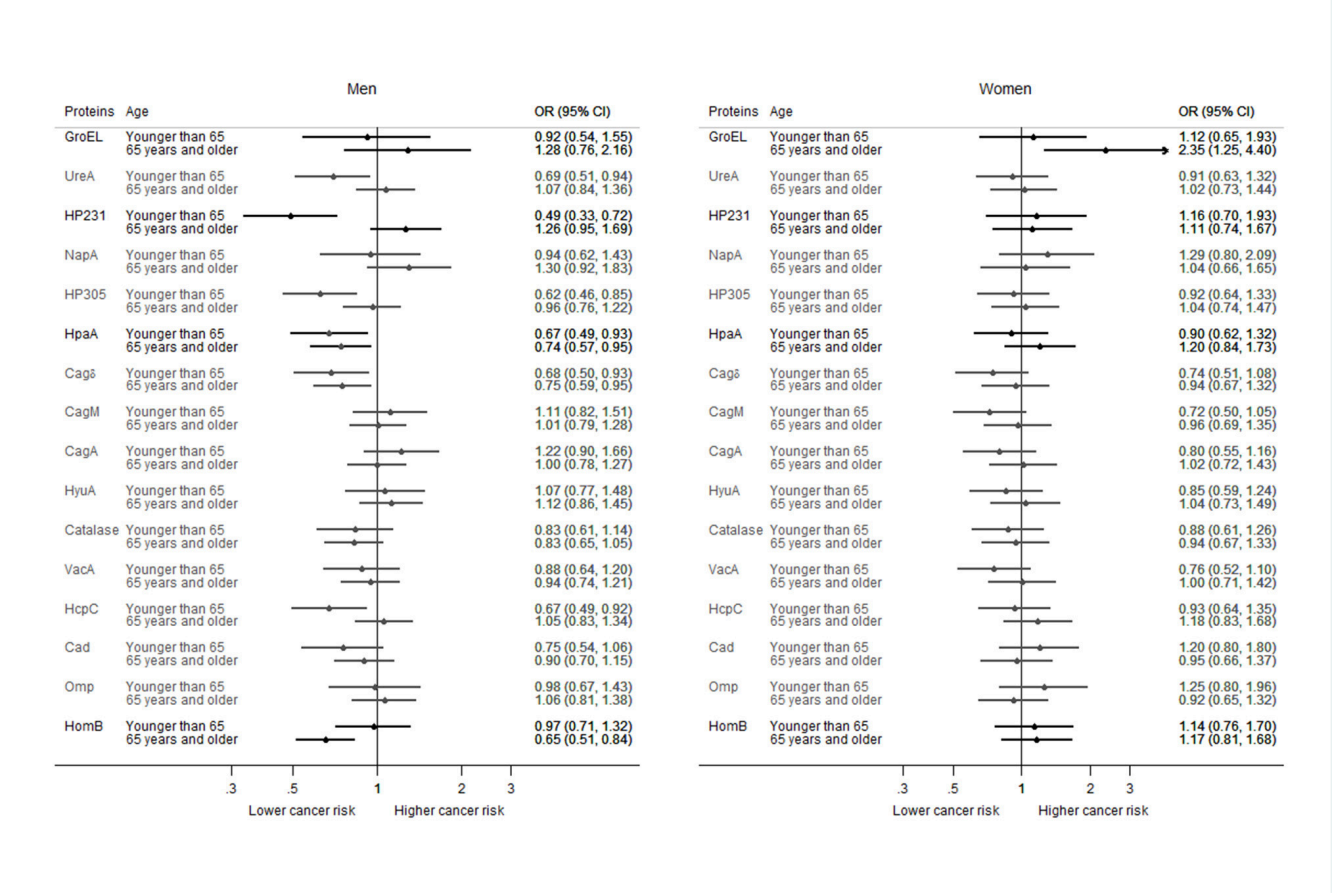
**Association of the seropositivity against each of the studied *H. pylori* protein with the risk of colorectal cancer among *H. pylori* seropositive participants, by gender and age-group**. ORs adjusted by education, age (as a continuous variable), smoking status, and family history of colorectal cancer. Province included as a random-effect term. Statistically significant interactions are highlighted in black.

Results from the analysis stratified by sex and age group suggested a different effect of *H. pylori* infection on the risk of CRC according to the combination of these characteristics. Women older than 64 years showed a nearly statistically significantly increased risk of CRC associated with *H. pylori* seropositivity (OR = 1.74; 95% CI: 0.99–3.08; *p*-value: 0.055), while no effect was observed for men of the same age group (OR = 1.18; 95% CI: 0.77–1.82; *p*-value: 0.452) or for younger women (OR = 0.95; 95% CI: 0.61–1.49; *p*-value: 0.834). On the other hand, in men under 65 years old *H. pylori* seropositivity was related to a lower CRC risk (OR = 0.61; 95% CI: 0.39–0.97; *p*-value: 0.036). The effect of seropositivity for individual proteins among infected subjects also showed differences by sex and age (Figure 3). Inverse associations were estimated for some proteins in men of the younger age group (<65 years old), while for both, men and women over 64 years most of the estimated OR were around unity, showing no effect. Interaction was statistically significant for GroEL, HP231, HpaA, and HomB. GroEL was associated with an increased CRC risk mainly in the age group over 64 years, being the association statistically significant only for women. HP231 showed a lower CRC risk in men under 65, a nearly significantly higher risk in men older than 64, and no effect in women. HpaA showed a lower CRC risk in men of both age groups and no effect in women. Lastly, HomB seropositivity was related to a lower risk of CRC only in men older than 64, with no effect in the other three groups.

## Discussion

This is one of the largest case-control studies published to date assessing the relation between CRC and *H. pylori* infection. Our results point to a lack of increased CRC risk associated with *H. pylori* infection. After controlling for the main known risk factors for CRC, neither *H. pylori* seropositivity, nor seropositivity against any of the analyzed proteins showed an increased risk of colon or rectum cancer. On the contrary, seropositivity against some *H. pylori* proteins, such as HP305, HpaA, and Cagδ was associated with a 15–21% reduced risk of CRC among infected subjects. When mutually adjusting for serostatus against other proteins, only Cagδ continued to show a statistically significant inverse association with CRC. Although this result could be due to chance, the dose-response trend observed for seroreactivity against this protein precludes us from categorically discarding a real association between seropositivity to this protein and a lower risk of CRC. To our knowledge, no clear physiopathological mechanism has been described to explain a possible protective role of Cagδ positive *H. pylori* infection in the development of CRC. Cagδ is one of the proteins forming the cag Type IV Secretion System of *H. pylori*. Integrity of this system has been implicated in several mechanisms favoring gastric carcinogenesis, but the specific function of Cagδ has not been fully elucidated. If our results were replicated in other studies, in depth research on the functions of Cagδ would be warranted.

Interestingly, no increased risk was associated with seropositivity to CagA (cytotoxin-associated gene A). This protein is a cytotoxin injected by *H. pylori* into the epithelial cells of the host that has been implicated in gastric carcinogenesis. Therefore, this finding further supports a lack of association of *H. pylori* infection with an increased risk of CRC or, in case of such an association to exist, the involvement of other carcinogenic mechanisms different from those mediated by CagA cytotoxicity.

Seropositivity against GroEL, a protein pertaining to the group of chaperons that has been related to gastric and CRC in some previous studies (Gao et al., 2009; Epplein et al., 2013; Murphy et al., 2015), was associated with an increased CRC risk in infected subjects in our sample when adjusting by serostatus against other proteins. This result should be interpreted cautiously, given that this is a highly conserved protein and serological analysis may have lower specificity due to cross-reaction with the corresponding proteins of other species. On the other hand, this result could be considered as supporting of a role of infections in general in the development of CRC.

Analyses stratified by age and sex showed some indications for a different association of the infection with the risk of CRC. *H. pylori* seropositivity was associated with a 74% higher odds of CRC in older women (4% increased risk for each additional seropositivity) and with a 39% lower risk in younger men (8% decreased risk for each additional seropositivity). With respect to the effect of seropositivity against individual proteins among *H. pylori* positive subjects, the decreasing risk associated with some of them was more evident in men and in younger age groups. Although age differences have been described on the relation of *H. pylori* infection and gastric cancer in some studies (Eybpoosh et al., 2015), there is not a general agreement in this point, neither a demonstration of such an effect for CRC. To the extent of our knowledge, no clear explanation has been largely accepted for this finding, and neither for a possible difference between men and women. However, this is a scarcely addressed issue and most of the published studies do not present their results stratified by age or sex, and consequently these factors are not generally included in sensitivity analyses of meta-analyses. An exception is the meta-analysis by Wu et al. (2013) that reported their results stratified by sex. A higher effect of *H. pylori* infection on the risk of CRC was estimated in women than in men, although the number of studies included was low and the authors concluded that there was no evidence for a different effect by sex. Our results could be taken into account to promote the realization of analyses stratified by these factors, in order to ascertain whether such an effect modification exits.

Given that differences in the predominant carcinogenesis pathways have been reported between right and left colon cancer, we evaluated whether the consequences of *H. pylori* infection differed by tumor site. Although statistical test for heterogeneity did not reveal significant differences, the observed inverse associations were statistically significant mainly for left colon cancer. This was true both for *H. pylori* seropositivity and for individual proteins among infected subjects. Therefore, we are cautious before rejecting a potential differential effect by tumor site.

In agreement with our results, two recently published nested case-control studies using also the multiplex serology technique to test *H. pylori* infection reported no statistically significant association between *H. pylori* seropositivity and colorectal, colon, or rectum cancer (Epplein et al., 2013; Blase et al., 2016). They did not find an association with CagA seropositivity either. However, Epplein et al. reported a higher risk of CRC for seropositivity against HcpC, HP305, VacA, HP231, and NapA, not encountered in our sample. These associations were further supported by a statistically significant dose-response pattern and were more evident for colon than for rectum cancer, although the low number of rectum cancer cases studied limits the interpretation of observed site-dependent differences, and they did not differentiate between right and left colon.

Regarding biological mechanisms that could be involved in a protective effect of *H. pylori* infection or of the infection with certain *H. pylori* strains over the development of CRC, several issues have to be mentioned. First of all, if *H. pylori* has been living with the human being for the last 50,000–70,000 years (Linz et al., 2007), one could assume that it has been playing a role in the ecosystem formed by human gastrointestinal tract microbiota. Therefore, in spite of the decrease of gastric cancer incidence largely attributed to the continuous reduction of *H. pylori* infection rates (secondary to the widespread use of antibiotics and the increase of the standard of living of most populations worldwide), the disappearance of *H. pylori* could be a cause of a detrimental alteration of the gastrointestinal microbiota (Kienesberger et al., 2016; Yap et al., 2016). The role of dysbiosis in human pathology is an area of increasing interest and the focus of multiple experimental and clinical research. With respect to CRC, several mechanisms have been proposed to explain the influence of gut microbiota on its development (Drewes et al., 2016). These include (1) a direct carcinogenic effect of individual bacterial species on the colonic epithelial cells, (2) an effect of the microbiota as a whole (which could modify the products derived from diet and metabolism, either in a beneficial or a harmful direction), or (3) mechanisms mediated by bacterial biofilm formation (such as favoring contact between bacteria and epithelial cells or causing a chronic inflammatory response). The disappearance of *H. pylori* infection would leave an ecological niche that, if occupied by harmful species could increase the risk of CRC (Butt et al., 2016; Drewes et al., 2016). Differences in the microbiota and in dietary habits between men and women and among age-groups in the population could therefore modulate the effect of *H. pylori* infection, which would be compatible with our finding of an effect modification associated with these factors.

Some limitations should be taken into account when interpreting our results. The case-control design of the study does not allow establishing or discarding causal relations. Also, residual confounding may remain due to lack of information about not measured potential confounding factors or to misclassification or insufficient accuracy of measured variables. In addition, a potential selection bias could be affecting our fully-adjusted results due to the lack of information on variables related to diet and BMI in a non-negligible proportion of subjects in our sample. However, in a sensitivity analysis done over the sample with complete data for these variables, the estimated ORs for *H pylori* seropositivity and for number of proteins seropositive did not change after including these variables into the model (data not shown). With respect to the measurement of the exposure, classification of infection based on serological tests does not differentiate between current and past infection. Besides, serological response depends not only on the exposure to the microorganisms but also on other factors such as the antigenicity of the different microbial proteins or the immune status of the host. Therefore, the interpretability of our results in terms of an effect directly attributed to the expression of proteins by *H. pylori* is limited. Particularly, a reverse causation secondary to immunosuppression potentially associated with cancer or oncological treatments cannot be ruled out. Lastly, the high *H. pylori* seroprevalence among controls in our sample, could lead to an underestimation of the OR.

This study has also several strengths. We included a high number of histologically confirmed incident colon and rectum cancer cases and recruited population-based controls. Furthermore, a reliable questionnaire was used which allowed to collect exhaustive information so that analyses were adjusted for many recognized CRC risk factors. In addition to the covariates included in the models presented here, we replicated all the analyses but adjusting also by the use of proton pump inhibitors and of non-steroidal anti-inflammatory drugs, and the results remained practically unaltered, which reinforces our confidence on the outcomes. Controlling for potential confounding factors has been determined as an important methodological tool in order to obtain unbiased estimates of the association between *H. pylori* infection and CRC (Zhang et al., 2012). In this same line, we did a crude analysis of our data, and the OR estimate for the association of *H. pylori* infection with CRC risk was 1.20, while the adjusted estimate was 0.85, supporting the importance of including potential confounding covariates in the statistical models and suggesting that some associations reported from studies done without such an adjustment could overestimate the effect. Besides, in our study statistical models included a random province-specific intercept term, which accounted for unexplained heterogeneity across different regions. The relatively high number of cases of rectum cancer included in our sample is other strength of the study, given that rectum cancer cases have frequently been underrepresented in other epidemiological studies in the field. In addition, we evaluated potential interactions by age, sex, and education. Finally, we used an emerging technology to measure the serological response against a wide range of *H. pylori* proteins, including some of the more widely recognized virulence factors, such as CagA, VacA, UreA, and Catalase.

To our knowledge, this is the first study that evaluates the association between *H. pylori* infection and CRC in Spain. Our results suggest that *H. pylori* seropositivity is not associated with a higher risk of colon or rectum cancer in the studied population. Antibody seroreactivity to CagA, highly associated with non-cardia gastric cancer, did not represent an increased risk either. On the contrary, we identified seropositivity against three individual proteins, mainly Cagδ, as markers of a reduced risk of CRC within this population, characterized by a high *H. pylori* seroprevalence. Our results point at a possible difference between sexes and age groups on the role of serological response against *H. pylori* infection either as a risk factor for or as a marker of CRC risk. If this finding was confirmed, differences in outcomes among published studies could be attributed in part to different population characteristics in terms of age and sex. Lastly, no statistical heterogeneity was identified in the effect of the infection among tumor sites, although some indications of a decreased risk for left colon cancer were observed.

## Ethics statement

The study protocol was approved by the Ethical Review Board of each participating center, and the study was carried out in accordance with their recommendations. All subjects gave written informed consent in accordance with the Declaration of Helsinki.

## Author contributions

NF analyzed the data, interpreted the results, and drafted the manuscript. NA, BP, EB, and MP planned and conducted the study, analyzed the data, interpreted the results, and reviewed the manuscript. AM, JB, MiP, TW, and BR performed serological assays, analyzed the data, interpreted the results, and reviewed the manuscript. VM, VMa, TD, JJ, JC, AT, IR, RP, AnT, MC, RO, IG, PL, AC, GC, MK, SS, and RC planned and conducted the study, interpreted the results and reviewed the manuscript. All authors have approved the version to be published and agree to be accountable for all aspects of the work.

### Conflict of interest statement

The authors declare that the research was conducted in the absence of any commercial or financial relationships that could be construed as a potential conflict of interest.
