# Supplementary material for: Helicobacter pylori Antibody Reactivities and Colorectal Cancer Risk in a Case-control Study in Spain
Source: Front Microbiol. 2017 May 29;8:888. doi: 10.3389/fmicb.2017.00888 (PMC5447227; doi:10.3389/fmicb.2017.00888)
Supplement: Supplementary file 1 [file Table1.docx]

Supplementary Material

***Helicobacter pylori* Antibody Reactivities and Colorectal Cancer Risk in a Case-control Study in Spain**

Nerea Fernández de Larrea-Baz*, Angelika Michel, Beatriz Romero, Beatriz Pérez-Gómez, Victor Moreno, Vicente Martín, Trinidad Dierssen-Sotos, José J. Jiménez-Moleón, Jesús Castilla, Adonina Tardón, Irune Ruiz, Rosana Peiró, Antonio Tejada, María D. Chirlaque, Julia A. Butt, Rocío Olmedo-Requena, Inés Gómez-Acebo, Pedro Linares, Elena Boldo, Antoni Castells, Michael Pawlita, Gemma Castaño-Vinyals, Manolis Kogevinas, Silvia de Sanjosé, Marina Pollán, Rosa del Campo, Tim Waterboer and Nuria Aragonés

*** Correspondence:** Nerea Fernández de Larrea: nfernandez@externos.isciii.es

**Supplementary Table 1**. *H. pylori* proteins and cut-offs used for serostatus classification. All the proteins were expressed from *H. pylori* strain 26695, except GroEL, from strain G27 and HomB from strain J99. Criteria for choosing proteins for the assay were: known surface exposure and immunogenicity in two-dimensional immunoblot analyses (UreA, HP231, NapA, HpaA, CagA, Catalase and VacA), serologic association with gastric cancer (GroEL, HyuA, Cad, HcpC and Omp) and/or with gastric ulcer (HP305 and CagM), and specific recognition in *H. pylori*-positive sera (Cagδ and CagM). MFI: Median reporter Fluorescence Intensity. ORF: Open Reading Frame.

| ***H. pylori* proteins** | | **Cut-off (MFI)** |
| --- | --- | --- |
| **Short name (ORF no.)** | **Full name** |  |
| GroEL (HP10) | Chaperonin GroEL | 100 |
| UreA (HP73) | Urease alfa subunit | 661 |
| HP231 (HP231) | Hypothetical protein | 100 |
| NapA (HP243) | Neutrophil activating protein (bacterioferritin) | 100 |
| HP305 (HP305) | Hypothetical protein | 100 |
| HpaA (HP410) | Neuraminyl lactose-binding hemagglutinin homolog | 246 |
| Cagδ (HP522) | *cag* pathogenicity island protein δ | 717 |
| CagM (HP537) | *cag* pathogenicity island protein M | 100 |
| CagA (HP547) | Cytotoxin-associated antigen A | 3,338 |
| HyuA (HP695) | Hydantoin utilization protein A | 260 |
| Catalase (HP875) | Catalase | 976 |
| VacA (HP887) | Vacuolating cytotoxin | 570 |
| HcpC (HP1098) | Conserved hypothetical secreted protein - paralogue HcpA | 100 |
| Cad (HP1104) | Cinnamyl-alcohol dehydrogenase ELI3-2 | 140 |
| Omp (HP1564) | Outer membrane protein | 809 |
| HomB (-) | *H. pylori* outer membrane protein B | 525 |
