# Supplementary material for: Helicobacter pylori Antibody Reactivities and Colorectal Cancer Risk in a Case-control Study in Spain
Source: Front Microbiol. 2017 May 29;8:888. doi: 10.3389/fmicb.2017.00888 (PMC5447227; doi:10.3389/fmicb.2017.00888)
Supplement: Supplementary file 2 [file Table2.docx]

Supplementary Material

***Helicobacter pylori* Antibody Reactivities and Colorectal Cancer Risk in a Case-control Study in Spain**

Nerea Fernández de Larrea-Baz*, Angelika Michel, Beatriz Romero, Beatriz Pérez-Gómez, Victor Moreno, Vicente Martín, Trinidad Dierssen-Sotos, José J. Jiménez-Moleón, Jesús Castilla, Adonina Tardón, Irune Ruiz, Rosana Peiró, Antonio Tejada, María D. Chirlaque, Julia A. Butt, Rocío Olmedo-Requena, Inés Gómez-Acebo, Pedro Linares, Elena Boldo, Antoni Castells, Michael Pawlita, Gemma Castaño-Vinyals, Manolis Kogevinas, Silvia de Sanjosé, Marina Pollán, Rosa del Campo, Tim Waterboer and Nuria Aragonés

*** Correspondence:** Nerea Fernández de Larrea: nfernandez@externos.isciii.es

**Supplementary Table 2**. Clinico-pathological characteristics of cases

| Variable | All  (N=1,488) | Colon  (N=910) | Rectum  (N=556) |
| --- | --- | --- | --- |
| Tumor site |  |  |  |
| Cecum | 127 (9%) | 127 (14%) | - |
| Ascending colon | 166 (11%) | 166 (18%) | - |
| Hepatic flexure | 48 (3%) | 48 (5%) | - |
| Transverse colon | 61 (4%) | 61 (7%) | - |
| Splenic flexure | 35 (2%) | 35 (4%) | - |
| Descending colon | 81 (5%) | 81 (9%) | - |
| Sigmoid colon | 391 (26% | 391 (43%) | - |
| Rectosigmoid segment | 84 (6%) | - | 84 (15%) |
| Rectum | 472 (32%) | - | 472 (85%) |
| Not specified | 23 (2%) | 1 (0%) | 0 (0%) |
| Histological type |  |  |  |
| Adenocarcinoma | 1,337 (90%) | 807 (89%) | 514 (92%) |
| Mucinous adenocarcinoma | 94 (6%) | 78 (9%) | 14 (3%) |
| Signet ring adenocarcinoma | 9 (1%) | 8 (1%) | 1 (0%) |
| Squamous carcinoma | 1 (0%) | 0 (0%) | 1 (0%) |
| Medullary carcinoma | 1 (0%) | 1 (0%) | 0 (0%) |
| Undifferentiated carcinoma | 3 (0%) | 3 (0%) | 0 (0%) |
| Other | 13 (1%) | 5 (1%) | 7 (1%) |
| Not specified/in situ | 30 (2%) | 8 (1%) | 19 (3%) |
| Blood collection moment |  |  |  |
| Prior/concomitant to treatment | 525 (35%) | 385 (42%) | 133 (24%) |
| First 2 months after treatment | 235 (16%) | 168 (18%) | 65 (12%) |
| >2 months after treatment | 379 (25%) | 160 (18%) | 215 (39%) |
| Missing | 349 (23%) | 197 (22%) | 143 (26%) |
| Initial treatment |  |  |  |
| Surgery | 1,040 (70%) | 805 (88%) | 223 (40%) |
| Chemotherapy | 157 (11%) | 33 (4%) | 122 (22%) |
| Chemo-radiotherapy | 100 (7%) | 1 (0%) | 99 (18%) |
| Radiation therapy | 74 (5%) | 5 (1%) | 69 (12%) |
| Missing | 117 (8%) | 66 (7%) | 43 (8%) |
