# Supplementary material for: Helicobacter pylori Antibody Reactivities and Colorectal Cancer Risk in a Case-control Study in Spain
Source: Front Microbiol. 2017 May 29;8:888. doi: 10.3389/fmicb.2017.00888 (PMC5447227; doi:10.3389/fmicb.2017.00888)
Supplement: Supplementary file 3 [file Table3.docx]

Supplementary Material

***Helicobacter pylori* Antibody Reactivities and Colorectal Cancer Risk in a Case-control Study in Spain**

Nerea Fernández de Larrea-Baz*, Angelika Michel, Beatriz Romero, Beatriz Pérez-Gómez, Victor Moreno, Vicente Martín, Trinidad Dierssen-Sotos, José J. Jiménez-Moleón, Jesús Castilla, Adonina Tardón, Irune Ruiz, Rosana Peiró, Antonio Tejada, María D. Chirlaque, Julia A. Butt, Rocío Olmedo-Requena, Inés Gómez-Acebo, Pedro Linares, Elena Boldo, Antoni Castells, Michael Pawlita, Gemma Castaño-Vinyals, Manolis Kogevinas, Silvia de Sanjosé, Marina Pollán, Rosa del Campo, Tim Waterboer and Nuria Aragonés

*** Correspondence:** Nerea Fernández de Larrea: nfernandez@externos.isciii.es

**Supplementary Table 3**. Association between potential confounding factors and *H. pylori* seropositivity in controls. HP -: Serology positive to less than 4 *H. pylori* proteins; HP +: Serology positive to at least 4 *H. pylori* proteins; BMI: Body mass index; CRC: Colorectal cancer; METS: Metabolic Equivalent Units. *Alcohol intake categories (based on g/d of ethanol intake): Light: ≤6; Upper recommended limit: women >6 - ≤12 and men >6 - ≤24; Abundant: women: >12 - ≤24 and men >24 - ≤60; Heavy/Very heavy: women >24 and men >60. **Values represent mean and standard deviations. For continuous variables, analysis was done over participants with complete information: 266 non-infected controls and 1889 infected controls for BMI; 266 non-infected controls and 1950 infected controls for diet variables; all non-infected controls and 2185 infected controls for physical activity measurement (METS). In the multivariable logistic regression analysis including variables with a p-value<0.10 in the univariable analysis presented in the table, sex and vegetables intake were associated to *H. pylori* seropositivity at a p-value<0.05.

| Variable | HP –  (N=309) | HP +  (N=2,186) | p-value |
| --- | --- | --- | --- |
| Sex |  |  |  |
| Male | 106 (34%) | 1,169 (53%) | <0.001 |
| Female | 203 (66%) | 1,017 (47%) |  |
| Race |  |  |  |
| White/Caucasian | 306 (99%) | 2,145 (98%) | 0.504 |
| Other | 3 (1%) | 39 (2%) |  |
| Education |  |  |  |
| No/incomplete primary school | 43 (14%) | 439 (20%) | <0.001 |
| Primary school | 84 (27%) | 807 (37%) |  |
| Secondary school | 105 (34%) | 580 (27%) |  |
| University degree | 77 (25%) | 360 (16%) |  |
| Smoking status |  |  |  |
| Never smoker | 142 (46%) | 957 (44%) | 0.219 |
| Former smoker | 93 (30%) | 761 (35%) |  |
| Current smoker | 74 (24%) | 459 (21%) |  |
| Past ethanol intake* |  |  |  |
| No drinker | 75 (24%) | 536 (25%) | 0.051 |
| Light | 63 (20%) | 392 (18%) |  |
| Upper recommended limit | 66 (21%) | 439 (20%) |  |
| Abundant | 49 (16%) | 391 (18%) |  |
| Heavy/Very heavy | 13 (4%) | 192 (9%) |  |
| Missing | 43 (14%) | 236 (11%) |  |
| BMI (Kg/m^2^) |  |  |  |
| <25 | 132 (43%) | 656 (30%) | <0.001 |
| 25-29.9 | 91 (29%) | 839 (38%) |  |
| ≥30 | 43 (14%) | 394 (18%) |  |
| Missing | 43 (14%) | 297 (14%) |  |
| CRC family history |  |  |  |
| No CRC family history | 273 (88%) | 1939 (89%) | 0.950 |
| Only 2^nd^ degree relatives | 8 (3%) | 57 (3%) |  |
| ≥1 first degree relative | 26 (8%) | 181 (8%) |  |
| Missing | 2 (1%) | 9 (0%) |  |
| Age (years)** | 59.9 [12.9] | 64.2 [11.4] | <0.001 |
| Total energy (cal/d)** | 1,862.4 [564.6] | 1,906.7 [556.3] | 0.105 |
| Fruit (g/d)** | 335.3 [214.0] | 360.0 [217.5] | 0.035 |
| Vegetables (g/d)** | 181.5 [117.1] | 195.5 [123.6] | 0.04 |
| Red/processed meat (g/d)** | 62.8 [42.8] | 61.8 [37.4] | 0.875 |
| Smoked cold meat/fish (g/d)** | 3.0 [6.7] | 3.3 [8.6] | 0.476 |
| Nuts and dried fruits (g/d)** | 9.4 [15.1] | 7.7 [13.0] | 0.008 |
| Dairy (g/d)** | 343.7 [188.3] | 367.8 [185.6] | 0.019 |
| Fiber (g/d)** | 22.1 [7.7] | 23.1 [9.3} | 0.229 |
| Calcium (mg/d)** | 898.5 [314.3] | 928.4 [305.0] | 0.041 |
| D vitamin (mcg/d)** | 2.7 [1.4] | 2.7 [1.5] | 0.858 |
| METS 2-12y before diagnosis** | 153.4 [271.4] | 155.0 [262.0] | 0.762 |
