# Supplementary material for: Helicobacter pylori Antibody Reactivities and Colorectal Cancer Risk in a Case-control Study in Spain
Source: Front Microbiol. 2017 May 29;8:888. doi: 10.3389/fmicb.2017.00888 (PMC5447227; doi:10.3389/fmicb.2017.00888)
Supplement: Supplementary file 4 [file Image1.PDF]

Supplementary Material

Helicobacter pylori Antibody Reactivities and Colorectal Cancer Risk in a Case-control Study in Spain

Nerea Fernández de Larrea-Baz*, Angelika Michel, Beatriz Romero, Beatriz Pérez-Gómez, Victor Moreno, Vicente Martín, Trinidad Dierssen-Sotos, José J. Jiménez-Moleón, Jesús Castilla, Adonina Tardón, Irune Ruiz, Rosana Peiró, Antonio Tejada, María D. Chirlaque, Julia A. Butt, Rocío Olmedo-Requena, Inés Gómez-Acebo, Pedro Linares, Elena Boldo, Antoni Castells, Michael Pawlita, Gemma Castaño-Vinyals, Manolis Kogevinas, Silvia de Sanjosé, Marina Pollán, Rosa del Campo, Tim Waterboer and Nuria Aragonés

* **Correspondence:** Nerea Fernández de Larrea: nfernandez@externos.isciii.es

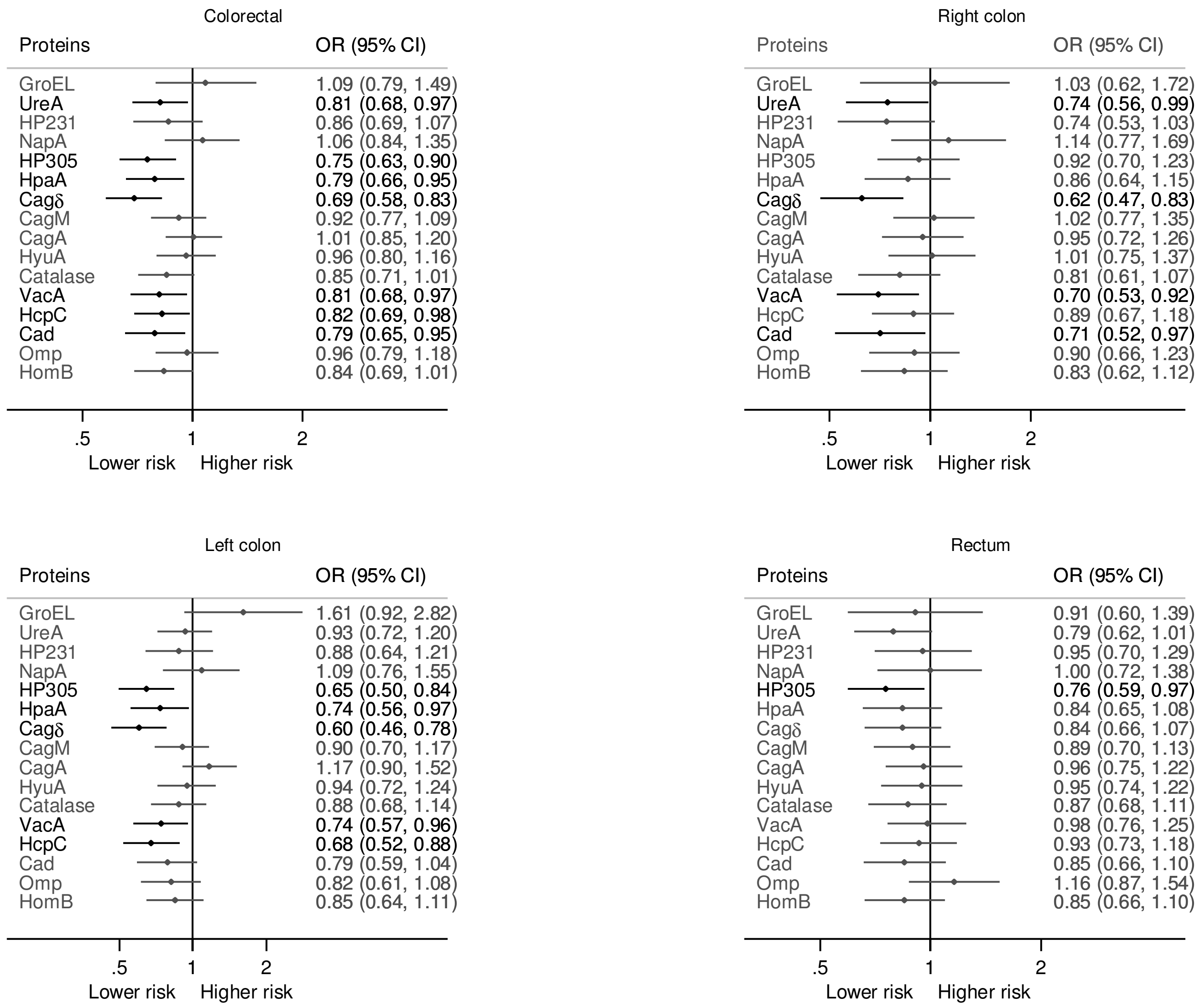

Supplementary Figure 1. Association of the seropositivity against each of the studied *H. pylori* proteins with the risk of colorectal cancer among infected participants (positive against 4 or more *H. pylori* proteins), overall and by tumor site. ORs from multinomial logistic regression mixed model adjusted by age, sex, education, family history of colorectal cancer, smoking status, body mass index, total energy intake, past ethanol intake, vegetables intake and red/processed meat intake; province included as a random-effect term. Statistically significant associations are highlighted in black. Analyses based on 1914 controls, 1031 colorectal, 265 right colon, 342 left colon and 409 rectal cancer cases with complete information for all the covariates.
